# Activated ERK Signaling Is One of the Major Hub Signals Related to the Acquisition of Radiotherapy-Resistant MDA-MB-231 Breast Cancer Cells

**DOI:** 10.3390/ijms22094940

**Published:** 2021-05-06

**Authors:** Anjugam Paramanantham, Eun Joo Jung, Se-IL Go, Bae Kwon Jeong, Jin-Myung Jung, Soon Chan Hong, Gon Sup Kim, Won Sup Lee

**Affiliations:** 1 Departments of Internal Medicine, Institute of Health Sciences and Gyeongsang National University Hospital, Gyeongsang National University College of Medicine, 90 Chilam-dong, Jinju 660-702, Korea; anju.udhay@gmail.com (A.P.); eunjoojung@gnu.ac.kr (E.J.J.); gose1@hanmail.net (S.-I.G.); 2 School of Veterinary and Institute of Life Science, Gyeongsang National University, 900 Gajwadong, Jinju 660-701, Korea; 3 Departments of Radiation Oncology, Institute of Health Sciences and Gyeongsang National University Hospital, Gyeongsang National University College of Medicine, 90 Chilam-dong, Jinju 660-702, Korea; blue129j@hanmail.net; 4 Departments of Neurosurgery, Institute of Health Sciences and Gyeongsang National University Hospital, Gyeongsang National University College of Medicine, 90 Chilam-dong, Jinju 660-702, Korea; gnuhjjm@gnu.ac.kr; 5 Departments of Surgery, Institute of Health Sciences and Gyeongsang National University Hospital, Gyeongsang National University College of Medicine, 90 Chilam-dong, Jinju 660-702, Korea; hongsc@gnu.ac.kr

**Keywords:** radiation-resistant, breast cancer, cell death, ERK, EMT, cancer stem cells (CSCs), PD98059

## Abstract

Breast cancer is one of the major causes of deaths due to cancer, especially in women. The crucial barrier for breast cancer treatment is resistance to radiation therapy, one of the important local regional therapies. We previously established and characterized radio-resistant MDA-MB-231 breast cancer cells (RT-R-MDA-MB-231 cells) that harbor a high expression of cancer stem cells (CSCs) and the EMT phenotype. In this study, we performed antibody array analysis to identify the hub signaling mechanism for the radiation resistance of RT-R-MDA-MB-231 cells by comparing parental MDA-MB-231 (p-MDA-MB-231) and RT-R-MDA-MB-231 cells. Antibody array analysis unveiled that the MAPK1 protein was the most upregulated protein in RT-R-MDA-MB-231 cells compared to in p-MDA-MB-231 cells. The pathway enrichment analysis also revealed the presence of MAPK1 in almost all enriched pathways. Thus, we used an MEK/ERK inhibitor, PD98059, to block the MEK/ERK pathway and to identify the role of MAPK1 in the radio-resistance of RT-R-MDA-MB-231 cells. MEK/ERK inhibition induced cell death in both p-MDA-MB-231 and RT-R-MDA-MB-231 cells, but the death mechanism for each cell was different; p-MDA-MB-231 cells underwent apoptosis, showing cell shrinkage and PARP-1 cleavage, while RT-R-MDA-MB-231 cells underwent necroptosis, showing mitochondrial dissipation, nuclear swelling, and an increase in the expressions of CypA and AIF. In addition, MEK/ERK inhibition reversed the radio-resistance of RT-R-MDA-MB-231 cells and suppressed the increased expression of CSC markers (CD44 and OCT3/4) and the EMT phenotype (β-catenin and N-cadherin/E-cadherin). Taken together, this study suggests that activated ERK signaling is one of the major hub signals related to the radio-resistance of MDA-MB-231 breast cancer cells.

## 1. Introduction

Breast cancer is one of the major causes of death due to cancer worldwide, especially in women [1]. For breast cancer, many therapies are available such as surgical resection, with or without lymph node dissection, radiation, and chemotherapy [2]. Radiation therapy is one of the important local regional therapies for breast cancer treatment [3]. Radiotherapy is applied for most breast cancer patients after surgical resection, but not all patients obtain the same benefits, because some of them suffer from a loco-regional relapse. Radio-resistance is the primary reason for this relapse [4].

Radio-resistance is a process in which the tumor cells or tissues adapt to radio therapy-induced damage [5] and survive irradiation (IR) [6,7]. Radiation can induce a DNA damage response (DDR), which causes cell cycle arrest and the induction of DNA repair, even though the cells with more severe damage from the radiation are induced to undergo apoptosis. The DDR may help the cells survive the IR-induced DNA damage, eventually developing radio-resistance by increasing the DDR rate. In addition, repopulation, hypoxic tumor areas, and cancer stemness are involved in radio-resistance. In DDR to IR and cancer stemness, several signaling pathways are reportedly involved: phosphatidylinositol 3-kinase (PI3K), mitogen-activated protein kinase (MAPK), SIRT pathways, Wnt/β-catenin signaling, IL22RA1/STAT3 signaling, and sonic Hedgehog signaling [8]. In addition to the suggested signaling pathways, IR resistance comprises the involvement of a large number of other proteins and their pathways [9,10]. Particularly, the radiation-induced ERK activation allows cancer cells to overcome the G2/M phase, which is considered the most vulnerable phase during IR, thereby causing radio-resistance [11,12,13].

MAPK pathways are key signaling pathways involved in the regulation of normal cell proliferation, survival, and differentiation. Aberrant regulation of the MAPK pathways contributes to the development of cancer; particularly, the extracellular signal-regulated kinase (ERK) is crucially involved in cancer cell proliferation, survival, and metastasis [14]. ERK consists of the p44 ERK1 and p42 ERK2. The ERK is the only known substrate of MEK. MEK1/2 activates ERK through dual tyrosine and threonine phosphorylation [15]. Thus, blocking the ERK pathway has proved to be an efficient mechanism to force cells into a cell death pathway. To potentiate the anti-tumoral effects of various cytotoxic agents, many trials of MEK1/2 pharmacological inhibitors (PD98059 [16], UO0126 [17], and PD184352 [18]) have been used. In addition, recent studies have shown the synergetic effect of the MEK/ERK inhibitor and radiation therapy [19].

Radio-resistant MDA-MB-231 breast cancer cells (RT-R-MDA-MB-231 cells) are reported to have a high proliferation rate, metastatic activity, and adhesion to endothelial cells compared with the parental MDA-MB-231 (p-MDA-MB-231) breast cancer cell line. RT-R-MDA-MB-231 cells harbor increased expressions of cancer stem cell (CSC) markers and the epithelial–mesenchymal transition (EMT) phenotype [8]. We hypothesized that there is a key altered signaling (driving oncogenic signaling) involved in developing RT-R-MDA-MB-231 cells. Here, we performed antibody microarray analysis to identify the hub proteins involved in the radio-resistance of RT-R-MDA-MB 231 cells by comparing p-MDA-MB-231 and RT-R-MDA-MB-231 cells because antibody microarray analysis is one of the technologies used for high-throughput protein characterization and discovery [10]. Antibody array analysis is used to measure the expression level of proteins between two different samples [9].

This study was designed to decipher the proteomic differences between p-MDA-MB-231 and RT-R-MDA-MB-231 cells, enabling us to corroborate our findings at the molecular level. In addition, we also aimed to investigate the importance of the key altered signaling in the reversal of radio-resistance and the regulation of the CSC and EMT phenotypes that are strongly associated with radio-resistance.

## 2. Results

### 2.1. Proteomic Profiling of RT-R-MDA-MB-231 Cell Lines

**Figure 1:**
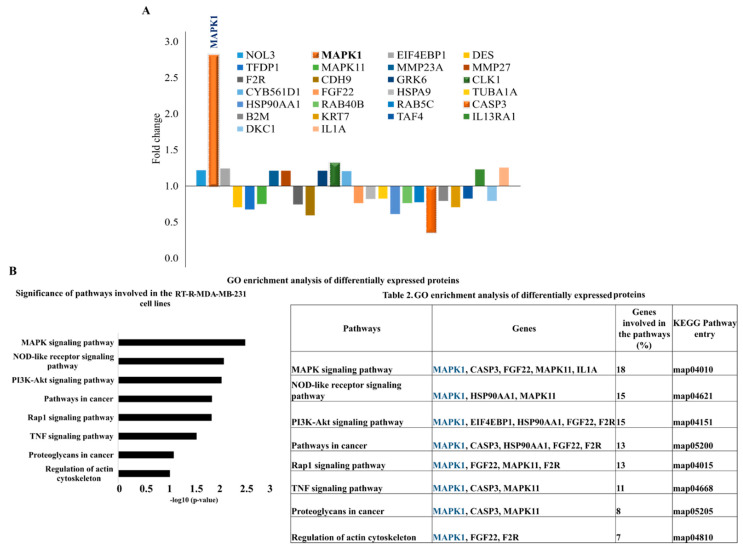
Antibody array analysis of radiation-resistant MDA-MB 231 breast cancer cells (RT-R-MDA-MB-231 cells). (**A**) Graphical representation of differentially expressed proteins with respect to the fold change in RT-R-MDA-MB-231 cells compared to parental MDA-MB-231 (p-MDA-MB-231) cells. (**B**) Gene ontology enrichment analysis of differentially expressed proteins in RT-R-MDA-MB-231 cells by KEGG analysis. GO enrichment analysis showed that MAPK1 is related to all the suggested signaling pathways involved in the radio-resistance of RT-R-MDA-MB-231 cells (Table 1).

To determine the key altered expressions of proteins involved in the radio-resistance of RT-R-MDA-MB 231 cells, we performed and analyzed antibody microarrays to assess the difference in protein expressions between p-MDA-MB-231 and RT-R-MDA-MB-231 cells. The internal normalization ratio (INR) was kept as INR > 1.0 and INR < 1.0. With respect to this value, we selected around 10 upregulated proteins and 16 downregulated proteins, which are specified in Figure 1A. The highly expressed proteins included mitogen-activated protein kinase 1 (MAPK1), which exhibited about a 2.81-fold increase compared to the p-MDA-MB-231 cells. Next to MAPK1, the highly expressed protein was dual-specificity protein kinase CDC-like kinases (CLK1), which exhibited a 1.32-fold increase.

Among the 16 downregulated proteins, caspase 3 was the most downregulated in the RT-R-MDA-MB-231 cells, which is suggested as one of the mechanisms for RT resistance [20]. Figure 1A shows a graphical representation of the proteins concerning the fold change. These findings suggested that the upregulation of MAPK1, CLK1, and FGF22 and the downregulation of caspase 3 might be involved in the acquisition of radio-resistant MDA-MB-231 cells.

### 2.2. MAPK1 Is the Most Important Signaling Pathway in Acquiring Radio-Resistant RT-R-MDA-MB-231 Cells

**Figure 2:**
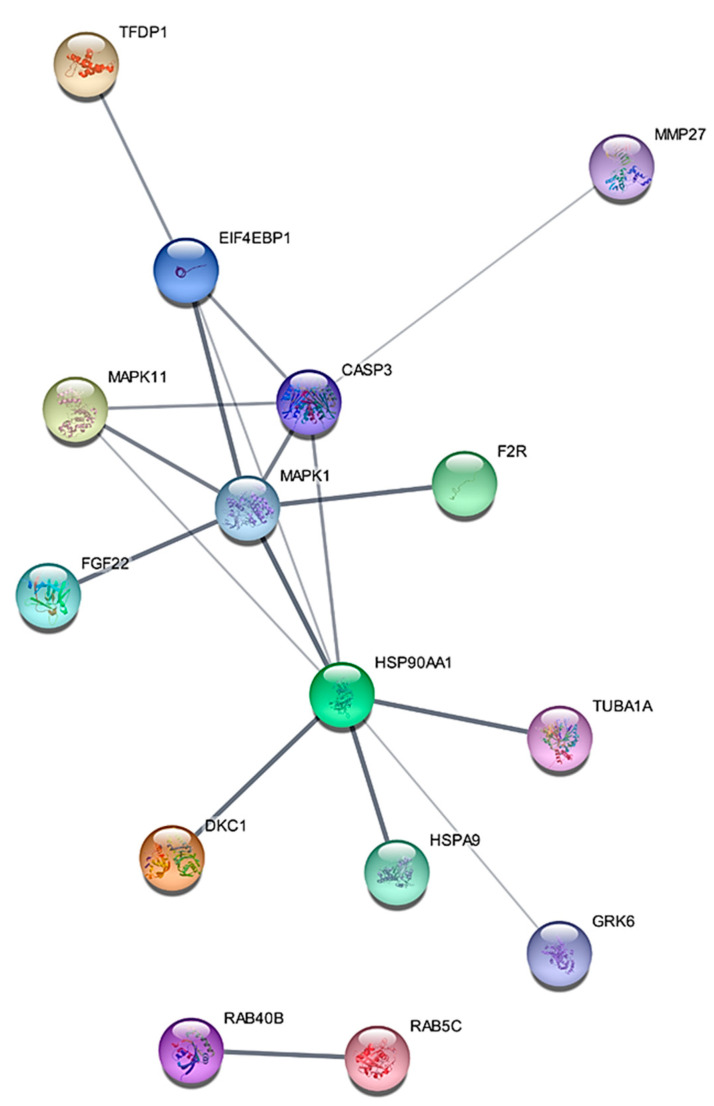
STRING (Cytoscape 3.6) analysis showed the protein–protein interaction (PPI) network of 13 proteins such as MAPK1, CASP3, FGF22, MAPK11, HSP90AA1, and F2R. They are involved in MAPK signaling, NOD-like receptor signaling, PI3K-Akt signaling, and Pathways in cancer in RT-R-MDA-MB-231 cells. The lines connecting the proteins depict “known” or “predicted” interactions. The thickness of the line corresponds to the strength of the interaction between the proteins. A total of 30 edges (protein–protein relationships) were discovered from 22 expected edges.

Gene ontology (GO) enrichment analysis of differentially expressed proteins was carried out with the use of KEGG (Kyoto Encyclopedia of Genes and Genomes) pathway analysis. The KEGG pathway analysis showed that the most significant pathway involved in the RT-R-MDA-MB-231 cells was the MAPK1 signaling pathway (Figure 1B). GO enrichment analysis suggested that MAPK1 is the most important signaling pathway in acquiring radio-resistant RT-R-MDA-MB-231 cells. The differentially expressed proteins were interrogated using the STRING database for the protein–protein interaction network analysis. String analysis of the protein–protein interaction (PPI) network generated an interconnected protein network with a medium confidence level of 0.04, which created a single module. The PPI network analysis of differentially expressed proteins showed a single module with 15 proteins such as MAPK1, CASP3, FGF22, MAPK11, HSP90AA1, and F2R. They are involved in MAPK signaling, NOD-like receptor signaling, PI3K-Akt signaling, and Pathways in cancer. The highly increased MAPK1 is related to all the suggested pathways. In addition, this module revealed that MAPK1 harbored a direct protein–protein interaction with caspase 3, which is crucial in inducing programmed cell death type 1 (apoptosis) (Figure 2). These findings support MAPK1 as being one of the important proteins involved in the acquisition of radio-resistant MDA-MB-231 cells.

### 2.3. ERK Signaling Was Important in the Cell Survival of RT-R-MDA-MB-231 Cells, and the Inhibition of MEK/ERK Signaling Reversed the Radio-Resistance of MDA-MB-231 Cells

**Figure 3:**
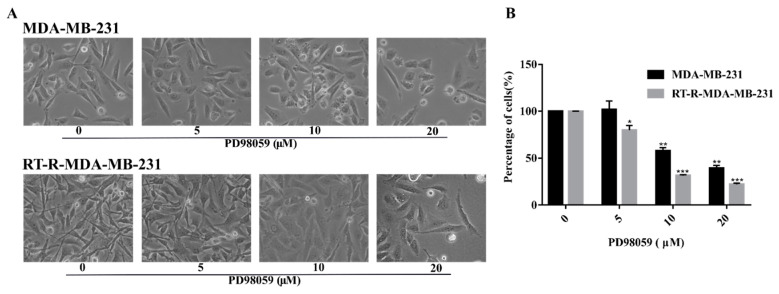
A MEK/ERK inhibitor (PD98059) inhibited the proliferation and promoted cell death in both p-MDA-MB-231 and RT-R-MDA-MB-231 cells. (**A**) p-MDA-MB-231 and RT-R-MDA-MB-231 cells were treated with PD98059 at the given concentrations. Morphological analyses were performed under a light microscope at 48 h. MEK/ERK inhibition resulted in significant morphological changes in both p-MDA-MB-231 and RT-R-MDA-MB-231 cells with a loss of cell integrity, as well as the reduction in cell population compared to the untreated intact cells (magnification, ×200; scale bar, 200 μm). (**B**) Cell viability was determined via MTT assay. The graph represents the % of viable cells after MEK/ERK inhibition. The values are expressed as mean ± standard deviation (SD) (*n* = 5) (* *p* < 0.05 vs. each control; ** *p* < 0.01 vs. each control; *** *p* < 0.005 vs. each control).

To investigate the inhibitory effect of a MEK/ERK inhibitor, PD98059, in RT-R-MDA-MB-231 cells, we performed cell viability assays in p-MDA-MB-231 cells and RT-R-MDA-MB-231 cells. Morphological analysis (Figure 3A) revealed that the RT-R-MDA-MB-231 cells were highly proliferative compared to the p-MDA-MB-231 cells even in the low dose of PD98059-treated cells (less than 20 μM). However, the proliferation rate of RT-R-MDA-MB-231 cells was not higher when they were treated with 20 μM of PD98059. The MTT assay showed that the anti-cancer effect of PD98059 was greater on RT-R-MDA-MB-231 cells than on p-MDA-MB-231 cells (Figure 3B). Morphological analysis (Figure 3A) also revealed that more cell deaths and cellular collapse were observed in RT-R-MDA-MB-231 cells than in p-MDA-MB-231 cells. In addition, there was a difference in morphology of the cell death between p-MDA-MB-231 cells and RT-R-MDA-MB-231 cells (Figure 3A). These findings suggested that ERK signaling should be important in the cell survival of RT-R-MDA-MB-231 cells, and that the inhibition of ERK signaling might reverse the radio-resistance of MDA-MB-231 cells.

### 2.4. Inhibition of ERK Signaling Reversed the Radio-Resistance of RT-R-MDA-MB-231 Cells

**Figure 4:**
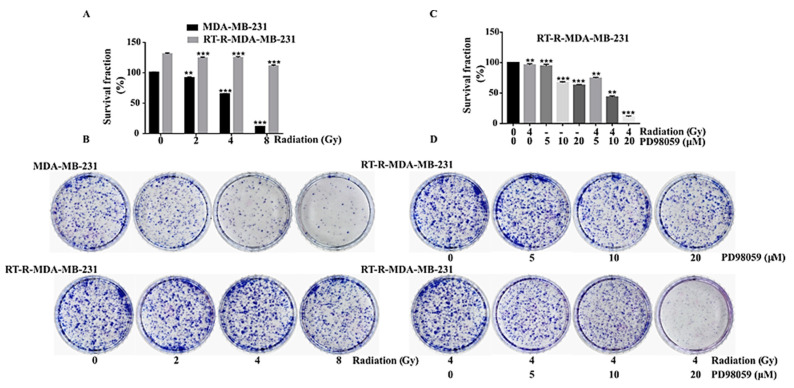
Clonogenic assay for effects of ERK inhibition on radio-resistance of RT-R-MDA-MB-231 cells. (**A**) Graphical representation of survival fraction of p-MDA-MB-231 cells and RT-R-MDA-MB-231 cells in % with the number of colonies after RT treatment. (**B**) Colony formation assay of RT-R-MDA-MB-231 cells and MDA-MB-231 cells. The cells were irradiated with different doses of RT (as indicated), they were grown for 2 weeks, and they were then stained with 0.1% Giemsa stain. Images were captured by a CCD (charge-coupled device) camera and the figures are representative of three independent experiments. (**C**) Graphical representation of RT-R-MDA-MB-231 cells survival fraction in % with the number of colonies after MEK/ERK inhibition with and without IR. (**D**) Colony formation assay of RT-R-MDA-MB-231 cells after MEK/ERK inhibition with and without IR and recorded as specified in (**B**). The values are represented as mean ± standard deviation (SD) (*n* = 5). ** *p* < 0.01; *** *p* < 0.005.

To explore the radio-sensitivity of both p-MDA-MB-231 and RT-R-MDA-MB-231 cells, we performed a colony formation assay. This revealed that RT-R-MDA-MB-231 cells were resistant to radiation (RT) until 4 Gy, whereas p-MDA-MB-231 cells were sensitive to RT treatment (Figure 4A,B). The colony number of RT-R-MDA-MB-231 cells was higher than that of p-MDA-MB-231 cells, which suggested that the RT-R-MDA-MB-231 cells were highly proliferative compared to p-MDA-MB-231 cells (Figure 4A,B). To investigate the correlation between activated ERK signaling and radio-resistance in RT-R-MDA-MB-231 cells, we performed an ERK inhibition test with a colony formation assay. As shown in Figure 4C,D, the inhibition of MEK/ERK (at 20 µM of PD98059) reversed the radio-resistance of RT-R-MDA-MB-231 cells. These findings support the importance of activated ERK signaling for the radio-resistance of RT-R-MDA-MB-231 cells.

### 2.5. Inhibition of ERK Signaling-Induced Necroptosis of RT-R-MDA-MB-231 Cells While It Induced the Apoptosis of p-MDA-MB-231 Cells

**Figure 5:**
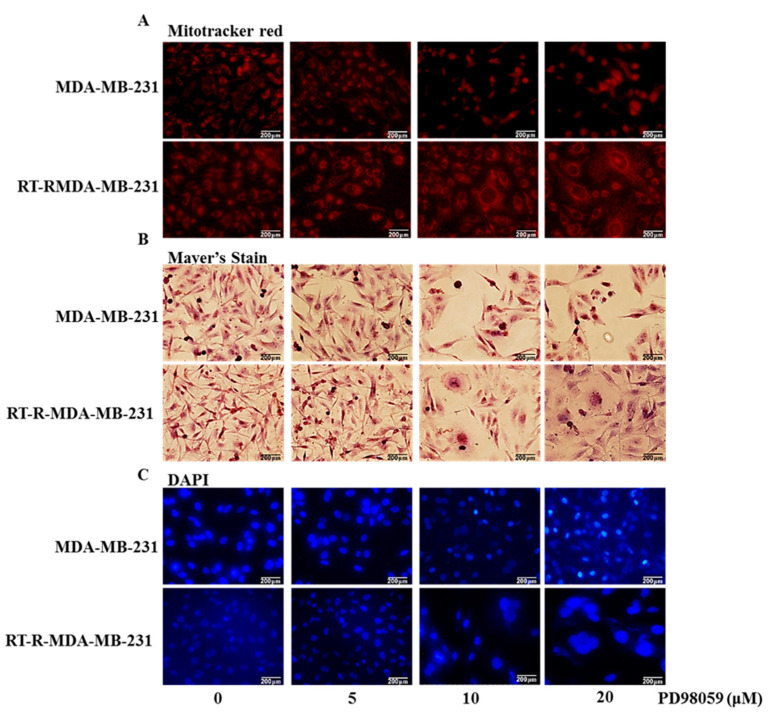
The difference in cell morphology between p-MDA-MB-231 and RT-R-MDA-MB-231 cells during ERK inhibition. Cells were seeded in 12-well plates with a 1 × 10^5^ cell/well density treated with the indicated concentrations of the MEK/ERK inhibitor (PD98059) for 24 h. (**A**) Mitochondrial morphology was analyzed with a fluorescent microscope after staining with MitoTracker (red). (**B**) Light microscopy of hematoxylin-stained cells showed the whole cell morphology of the MEK/ERK inhibitor-induced cell death. (**C**) Nuclear morphology analysis of the MEK/ERK inhibitor-induced cell death with DAPI staining. Results were confirmed by three independent experiments.

In Figure 3A, we found differences in the morphology between p-MDA-MB-231 cells and RT-R-MDA-MB-231 cells after ERK inhibition. To elucidate the differences in cell morphology between the two types of cells, we performed mitochondria staining, Mayer’s hematoxylin staining for the cell structure, and DAPI for the nucleus. MitoTracker^®^ Red staining is used to show the live time status of mitochondria [21]. The staining revealed that, with the treatment of the MEK/ERK inhibitor, mitochondrial fragmentation was seen in RT-R-MDA-MB-231 cells at the 24 h-inhibition of ERK signaling (Figure 5A). With the inhibition of ERK signaling, RT-R-MDA-MB-231 cells showed more fragmentation and swollen mitochondria than p-MDA-MB-231 cells did, suggesting that ERK inhibition contributes to the mitochondrial fission in RT-R-MDA-MB-231 cells. Mayer’s hematoxylin staining revealed that 24 h-MEK/ERK inhibition induced the cell swelling of nuclei and cytoplasm in RT-R-MDA-MB-231, while it induced the shrinkage of nuclei in the p-MDA-MB-231 cell (Figure 5B). These results were also confirmed with DAPI staining. The DAPI staining revealed a high level of nuclear swelling in RT-R-MDA-MB-231 cells treated with the MEK/ERK inhibitor, and it revealed nuclear fragmentation in p-MDA-MB-231 cells (Figure 5C). These results suggest that the ERK inhibition promotes cell death in both p-MDA-MB-231 and RT-R-MDA-MB-231 cells, but that the mechanisms for the cell death of the two cells were different.

### 2.6. ERK Inhibition Induced Caspase Activation and PARP-1 Cleavages in p-MDA-MB-231 Cells, While It Did Increase the Expression of Cyclophilin A (CypA) and AIF in RT-R-MDA-MB-231 Cells

**Figure 6:**
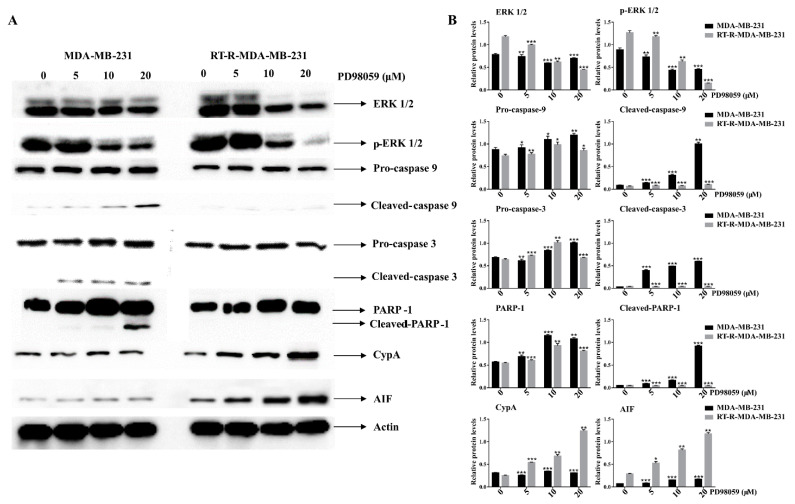
Effects of ERK inhibition on cell death-related proteins in both p-MDA-MB-231 and RT-R-MDA-MB-231 cells. Cells were seeded with a seeding density of 5 × 10^4^ cells and were pretreated with the MEK/ERK inhibitor (PD98059) for 48 h. The control cells were left untreated. The whole cell protein lysate was prepared and 30 μg of proteins was resolved in SDS-polyacrylamide gels. (**A**) Western blot analysis of various cell death-related proteins. (**B**) Densitometry analysis of the data in Western blot analysis by ImageJ software. The values were normalized against β-actin, and they are represented as mean ± standard deviation (SD) (*n* = 3). ** *p* < 0.01; *** *p* < 0.005 vs. the control group.

To molecularly confirm the difference in cell death between p-MDA-MB-231 and RT-R-MDA-MB-231 cells, we performed Western blot analysis. Figure 6 demonstrates that, in MDA-MB-231 cells, ERK inhibition induced the cleavage PARP-1 and caspase-3, which is a hallmark for caspase-dependent apoptosis, but that, in the RT-R-MDA-MB-231 cells, ERK inhibition induced AIF (apoptosis-inducing factor), which positively regulates the CypA protein, which is considered a biomarker of necroptosis [22]. These findings support ERK inhibition inducing the apoptosis of p-MDA-MB-231 and the necroptosis of RT-R-MDA-MB-231 cells.

### 2.7. ERK Inhibition Reduced the Expression of Cancer Stem Cell (CSC) Markers (CD44 and Oct ¾) and the EMT Phenotype, Which Is Closely Related to the Radio-Resistance of RT-R-MDA-MB-231 Cells

**Figure 7:**
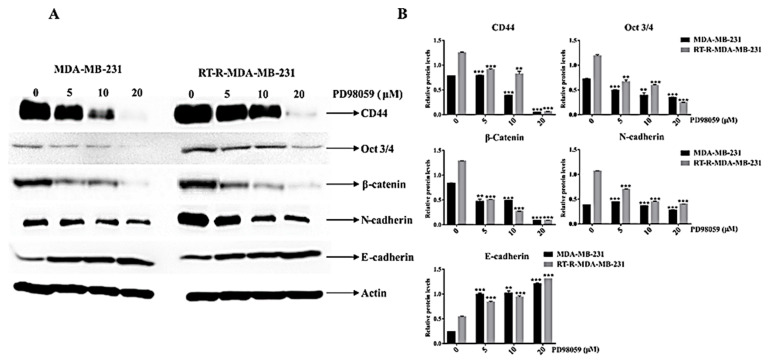
Effects of ERK inhibition on the expression of CSC markers (CD44 and Oct ¾), β-catenin, and EMT markers (E-cadherin and N-cadherin) in both p-MDA-MB-231 and RT-R-MDA-MB-231 cells. (**A**) Western blot analysis of CSC markers (CD44 and Oct ¾), β-catenin, and EMT markers (E-cadherin and N-cadherin). (**B**) Densitometry analysis of the data in Western blot analysis by ImageJ software. The values were normalized against β-actin, and they are represented as mean ± standard deviation (SD) (*n* = 5). ** *p* < 0.01; *** *p* < 0.005 vs. the control group.

It was reported that CSC markers and EMT phenotypes were highly expressed in RT-R-MDA-MB-231 cells compared to the p-MDA-MB-231 cells, and that their high expression was closely related to radio-resistance [8]. Here, we assessed the effect of ERK inhibition on the expression of CSC markers and the EMT phenotype on both MDA-MB-231 and RT-R-MDA-MB-231 cells. As previously reported, Western blot analysis revealed that RT-R-MDA-MB-231 cells showed a higher expression of CSC markers (CD44 and Oct 3/4) and EMT markers (N-cadherin and β-catenin) compared to the p-MDA-MB-231 cells (Figure 7A,B). ERK inhibition significantly suppressed the expression of CSC markers and the EMT phenotype in both p-MDA-MB-231 and RT-R-MDA-MB-231 cells (Figure 7A,B). In addition, the ERK inhibition more prominently suppressed the EMT phenotype of RT-R-MDA-MB-231 cells than p-MDA-MB-231 cells. These findings indicated that the ERK inhibition clearly suppressed the high expression of CSC markers and the EMT phenotype of RT-R-MDA-MB-231 cells that are reportedly associated with radio-resistance [5].

## 3. Material and Methods

### 3.1. Protein Array Analysis

The total proteins of p-MDA-MB-231 and RT-R-MDA-MB-231 cells were isolated with a radioimmunoprecipitation assay (RIPA) buffer, which contained 0.1% NP-40 and 0.1% sodium dodecyl sulfate in phosphate-buffered saline (PBS) containing a protease inhibitor cocktail (Sigma Aldrich, St. Louis, MO, USA). The expression profiling of proteins was analyzed by a Signaling Explorer Antibody Array (Ebiogen, Seoul, Korea).

### 3.2. Bioinformatics Analysis

The obtained proteins from the antibody array analysis were further submitted to DAVID (The Database for Annotation, Visualization, and Integrated Discovery). DAVID is an online tool that provides a biological understanding between two or more data sets of genes, and it can also be used to determine gene ontology (GO) in terms of biological processes and cellular processes. To determine the pathways involved in the identified genes, KEGG (Kyoto Encyclopedia of Genes and Genomes) (https://www.genome.jp/kegg/pathway.html, accessed on 20 October 2018) was employed. The selected genes were investigated for potential protein–protein interactions using STRING (Search Tool for the Retrieval of Interacting Genes) database version: 10.5 (https://string-db.org, accessed on 19 February 2021). For the display of protein interactions, selected proteins were uploaded into the STRING database and assessed using Cytoscape Software version Cytoscape_v3.7.1 (https://www.cytoscape.org, accessed on 19 February 2021). To access the interaction of the experimental data and to provide unambiguous comprehensive coverage, the online tool string was used.

### 3.3. Cell Culture

RT-MDA-MB-231 cells were established as previously described [8]. Briefly, MDA-MB-231 cells were fractionated with X-ray irradiation until a final dose of 50 Gy was reached. p-MDA-MB-231 and RT-MDA-MB-231 cells were cultured in RPMI-1450 medium supplemented with 10% heat-inactivated FBS and 1% penicillin/streptomycin. The cells were maintained at 37 °C in a 5% CO_2_ incubator. The cells were grown with 80% confluence and were treated with a MEK/ERK inhibitor (PD98059) dissolved in DMSO or DMSO alone.

### 3.4. Cell Viability Assay

We used a calorimetric assay, MTT (3-(4,5-dimethylthiazol-2-yl)-2,5-diphenyltetrazolium bromide), to analyze the cell viability. The cells were seeded in 24-well plates with a confluence of 1 × 10^5^ cells/well, they were treated with the MEK/ERK inhibitor, and they were maintained for 24 and 48 h at 37 °C in a 5% CO_2_ incubator. After incubation, 50 µL of MTT (0.5 mg in 1× PBS) was added to each well and incubated for about 2 h at 37 °C in a 5% CO_2_ incubator. The media were removed and the formazan crystals that formed in the live cells were dissolved with the 500 µL of DMSO. The solubilized formazan crystals were transferred to 96-well plates and the absorbance was read by an enzyme-linked immunosorbent assay (ELISA) reader at 540 nm. The cell viability was quantified in percentage, while vehicle-treated control cells were set at 100%.

### 3.5. Colony Formation Assay

P-MDA-MB-231 or RT-R MDA-MB-231 cells (1 × 10^3^ cells/well) were seeded in six-well plates, treated with the indicated doses of the MEK/ERK inhibitor, and maintained at 37 °C in a 5% CO_2_ incubator. The cells were irradiated with a given concentration, and the media were discarded after 24 h and replaced with fresh complete media every 2–3 days. After 14 days, the medium was discarded and the cells were washed with 1× PBS thrice. The colonies were fixed with absolute methanol for 10 min, stained with Giemsa staining solution, and then maintained at room temperature. The number of colonies was counted using ImageJ software.

### 3.6. Cytochemical Staining Methods

#### 3.6.1. Mitotracker Red Analysis

For mitochondrial morphology analysis, Mitotracker Red dye was used. The cells were seeded with a confluence of 1 × 10^5^ cell/well in 12-well plates, they were treated with the MEK/ERK inhibitor for 24 h, and they were washed with 1× PBS and then stained with 0.5 µL of Mitrotracker red in 500 µL of 1× PBS. The cells were incubated for 30 min in a 5% CO_2_ incubator at 37 °C. After incubation, the cells were viewed under a fluorescent microscope for the analysis of the live mitochondrial status after the treatment of a MEK/ERK inhibitor.

#### 3.6.2. Hematoxylin Staining

The cells were seeded in 12-well plates with a confluence of 1 × 10^5^ cells/well and were grown for 24 h with the MEK/ERK inhibitor at 37 °C in a CO_2_ incubator. After incubation, the cells were washed with 1× PBS and then fixed with 4% para-formaldehyde overnight. The fixed cells were washed thrice with 1× PBS for about 5 min per wash, they were stained with 200 µL of Mayer’s hematoxylin staining solution, and they were incubated for 20 min in the dark at room temperature. Then, the cells were washed thoroughly with 1× PBS, followed by 1 mL of 90% glycerol, and they were observed under a phase-contrast microscope.

#### 3.6.3. DAPI (4′,6-diamidino-2-phenylindole) Staining

For the nuclear morphological changes, DAPI staining was performed. The cells were seeded in 12-well plates at a density of 1 × 10^5^ cells/well with the treatment of the MEK/ERK inhibitor for about 24 h, they were washed with 1× PBS, and they were fixed overnight with 4% formaldehyde. After fixation, the cells were washed with 1× PBS thrice for about 5 min per wash. DAPI solution (0.5 µL) was added to the 500 µL of 1× PBS, which was incubated for 30 min at 37 °C with a 5% CO_2_ incubator. After incubation, the cells were washed with 1× PBS and were fixed with 90% glycerol in 1× PBS. The cells were viewed under a fluorescent microscope (Leica Microsystems GmbH, Wetzlar, Germany).

### 3.7. Western Blot Analysis

P-MDA-MB-231 and RT-MDA-MB-231 cells were seeded in 100 mm plates with a cell density of 2 × 10^6^ cells/plate. The cells were treated with the MEK/ERK inhibitor or DMSO as a vehicle control and were maintained for 48 h at 37 °C with a 5% CO_2_ incubator. After 48 h, the cells were harvested and transferred to 15 mL falcon tubes, and they were centrifuged for 5 min at 2000 rpm. The supernatant was discarded, and the tubes were centrifuged again to remove the residual supernatant. After complete removal of the supernatant, 500 µL of the 2X sample buffer containing 100 mM of Tris-Cl (pH 6.8), 4% (*w*/*v*) sodium dodecyl sulphate (SDS), 0.2% (*w*/*v*) bromophenol blue, and 200 mM of dithiothreitol was added. The protein lysates were collected in 1.5 mL Eppendorf tubes and kept at 100 °C for 10 min. The protein concentration was determined by the Bradford assay. In addition, 30 µg of the proteins was resolved in 8–12% SDS-PAGE and was transferred to a polyvinylidene difluoride membrane. After transfer, the membranes were blocked with 3% skimmed milk in Tris-buffered saline containing 1% Tween 20 (TBST) buffer for 30 min at room temperature, and they were incubated at 4 °C overnight with antibodies against actin (A5441, 1:10,000, Sigma-Aldrich, St. Louis, MO, USA), ERK (SC-94, 1:2000, Santa Cruz Biotechnology, Dallas, TX, USA), p-ERK (SC-7383, 1:2000, Santa Cruz Biotechnology), CypA (SC-134310, 1:2000, Santa Cruz Biotechnology), pro-caspase 9 (SC-56076, 1:2000, Santa Cruz Biotechnology), pro-caspase 3 (SC-7272, 1:2000, Santa Cruz Biotechnology), AIF (SC-55519, 1:1000, Santa Cruz Biotechnology), PARP-1 (SC-8007, 1:2000, Santa Cruz Biotechnology), CD44 (ab51037, 1:2000, Abcam, Cambridge, UK), β-catenin (SC-7199, 1:2000, Santa Cruz Biotechnology), Oct 3/4 (SC-5279, 1:2000, Santa Cruz Biotechnology), E-cadherin (ab1416, 1:2000, Abcam), and N-cadherin (ab76011, 1:2000, Abcam). After overnight incubation in primary antibodies, the membranes were washed with TBST buffer thrice for about 10 min per wash. Then, the membranes were incubated in horseradish peroxidase (HRP)-conjugated secondary antibody for 2 h at room temperature with 1:2000 dilution. The membranes were later washed with TBST buffer three times (10 min/wash) and were developed with ECL (electrochemiluminescence) solutions (Bio-Rad Laboratory, Hercules, CA, USA).

### 3.8. Statistical Analysis

All experiments were performed at least in triplicate, and all analyses were performed with the use of GraphPad Prism 7 software (GraphPad Software, San Diego, CA, USA). One-way ANOVA followed by the Newman–Keuls post hoc test was performed to compare various treatment groups. The data were presented as mean ± standard deviation (SD). A *p*-value <0.05 was considered statistically significant.

## 4. Discussion

Radiation therapy is one of the common and essential parts of breast cancer treatment. Around half of the cancer patients go through radiation therapy at some point in their treatment [23]. Ionizing radiation (IR) induces DNA damage through oxidative stress. The free OH radicals are capable of promoting single-stranded and double-stranded DNA breaks (SSB and DSB, respectively), which, in turn, triggers cell death [24]. Thus, cells develop IR resistance by counteracting the four ‘R’s,’ which is DNA damage by DNA repair, redistribution, repopulation, and reoxygenation through the activation of various pathways [25]. Several studies have described the role of irradiation in breast cancer pathways and the involvement of several proteins in the development of resistance against radiation, which we have listed in Table 2. Only a small number of studies have investigated mechanisms of acquired radio-resistance through the generation of radio-resistant cell lines, which tend to focus on a single specific pathway. Thus, it is paramount to focus on the signaling mechanism through the generation of the radio-resistant breast cancer cell line.

This study was designed to find the hub signaling involved in the RT resistance of RT-R-MDA-MB-231 cells and to investigate the importance of the hub signaling in the reversal of radio-resistance and the regulation of the CSC and EMT phenotype that is highly associated with radio-resistance. We found that ERK signaling was highly activated in RT-R-MDA-MB-231 cells compared to in p-MDA-MB-231 cells and that ERK signaling was essential for the survival of both p-MDA-MB-231 and RT-R-MDA-MB-231 cells. In addition, the RT resistance of RT-R-MDA-MB-231 cells was reversed by the inhibition of ERK signaling (Figure 4). Furthermore, we demonstrated that the activated ERK signaling was associated with cancer stemness and EMT phenotype (Figure 7). Considering all these findings, we can conclude that activated ERK signaling is one of the major hub signaling related to the acquisition of radio-resistant MDA-MB-231 cells.

Before concluding, we should discuss some questions. The first question would be whether activated ERK signaling is the main mechanism for the radio-resistance of MDA-MB-231 cells. In Figure 4 and Figure 7, the MEK/ERK inhibition test revealed that MEK/ERK inhibition induced cell death and suppressed the expression of CSC markers and the EMT phenotypes of both p-MDA-MB-231 cells and RT-R-MDA-MB-231 cells. This finding also suggested that ERK signaling is essential for the survival of MDA-MB-231 cells and that it may not be related to RT resistance per se. We also agree with the point. In addition, it was reported that other signaling pathways such as PI3K/Akt, and STAT or other anti-apoptotic proteins are important in the radio-resistance or radio-sensitivity of MDA-MB 231 cells (Table 2). However, there is some evidence to support that the ERK signaling is related to the RT-resistance of RT-R-MDA-MB-231 cells. Recent studies have depicted that the ERK1/2 activation prevails over the cell cycle arrest in the G2/M phase where cancer cells are susceptible to IR, thus inducing radio-resistance [5]. Another is that MDA-MB-231 cells are a triple-negative breast cancer cell line, which is known to have high CSC properties [49]. The third is that in our previous report, RT-R-MDA-MB-231 cells also showed an increased STAT 3 activity, which was reported to be related to cancer stemness and EMT, but that the inhibition of STAT 3 activity by the JNK inhibitor or Janus-activated kinase 2 (JAK2) inhibitor could not suppress the increased expression of CSC markers [50]. To solve this question, we carried out this study with antibody microarray analysis, which revealed that highly increased MAPK1 was enriched in all of the upregulated pathways of RT-R-MDA-MB-231 cells (MAPK signaling, NOD-like receptor signaling, PI3K-Akt signaling, and Pathways in cancer). In addition, the KEGG pathway enrichment analysis showed that all enriched pathways include MAPK1, and the PPI network analysis of differentially expressed proteins showed that MAPK1 could be related to the resistance of cell death. The third is that the inhibition of ERK signaling reversed RT resistance (Figure 4). The inhibition of ERK signaling was reported to increase the anti-cancer efficacy of RT [51,52]. This finding could support the reversal of RT resistance caused by the inhibition of ERK signaling. With all of these findings, we can say that activated ERK signaling is one of the main mechanisms for the radio-resistance of MDA-MB-231 cells.

The second point to discuss would be the relationship between ERK signaling and EMT, as well as the CSC phenotype of RT-R-MDA-MB-231 cells, because it is mentioned that several other signaling pathways such as JAK/STAT, Hedgehog, Wnt, Notch, PI3K/PTEN, and nuclear factor-κB (NF-κB) signaling pathways, compared to ERK signaling, are closely related to CSC properties [53,54,55], and the blocking of these pathways involved might be an effective way to target CSCs [55]. Even though it is not common, PRMT6-dependent CRAF/ERK signaling was reported to regulate CSC plasticity [56]. In addition, it was reported that the CSC properties-related signal is frequently complexed, and there is cross-talk between and among the mentioned various pathways [57]. In addition, the influence of ERK activity in regulating the CSC phenotype is also reported in gemcitabine-resistant pancreatic cells [58], cisplatin-resistant non-small cell lung cancer cells [59], and docetaxel and carboplatin-resistant ovarian cancer cells [60]. To determine the real cause of the activated ERK signaling of RT-R-MDA-MB-231 cells, we performed whole genome sequencing. In the study, we could not find any mutations on the linear line for the activation of ERK signaling (data not shown), such as EGFR, SOS, B-raf, Ras, or MEK. Regarding this question, we could not give the audience clear evidence. Therefore, further studies are required.

The third point to discuss is why the phenotype of cell death induced by ERK inhibition differed between p-MDA-MB-231 cells and RT-R-MDA-MB-231 cells, while ERK inhibition induced cell death and suppressed the increased expression of CSC markers and the EMT phenotype of both p-MDA-MB-231 cells and RT-R-MDA-MB-231 cells. We speculate that the reason could be that RT-R-MDA-MB-231 cells exhibit a decreased activity of caspase. It has been reported that the cancer cells harboring caspase defects frequently undergo necroptosis or necrosis instead of apoptosis when the death signal appears [61]. Initially, we thought that the defects in caspase 3 activity were the main cause that was associated with increased ERK signaling, which was revealed by string analysis of the PPI network. By whole genome sequencing, we also found that RT-R-MDA-MB-231 cells harbor a nonsynonymous single nucleotide mutation in CASP9 (Arg173His; rs2308950), which is known to be involved in the pathogenesis of various cancers (data not shown) [62,63]. We speculate that this mutation is also responsible for RT-R-MDA-MB-231 cells undergoing necroptosis during the inhibition of ERK signaling.

The fourth point to discuss would be the role of the other upregulated and downregulated proteins in RT-R-MDA-MB-231 cells. Although we could not discuss here all of the 26 proteins, recent studies have suggested that the inhibition of CLK1 also decreases cell proliferation [64]. CLK1 and FGF22 are oncogenes in cancer and their inhibition leads to the inhibition of breast cancer growth in cell culture and xenograft models [65,66]. This supports the possible contribution of upregulated CLK1 to the rapid growth of RT-R-MDA-MB-231 cells. The most downregulated protein, caspase 3, may also contribute to the radio-resistance of RT-R-MDA-MB-231 cells, by avoiding IR-induced apoptosis [67]. All of these findings suggest that the changes in the expression of proteins may be involved in the biological phenotype of RT-R-MDA-MB-231 cells. Regarding these points, further studies are warranted.

The fifth point to discuss would be the mechanisms driving the upregulation of ERK signaling in RT-R-MDA-MB-231 cells. As we know that radiotherapy works by damaging the DNA of cancer cells, our first thought was that the upregulation of ERK signaling would be related to some of the mutations in the Ras-Raf-MEK-ERK pathway. Therefore, we performed whole genome sequencing, but there was no additional mutation of ERFR, PI3K/Akt, Ras, Raf, MEK, or ERK molecules of MDA-MB-231 cells (data not shown). In this paper, we inhibited ERK signaling with PD98059, a non-adenosine triphosphate competitive MAPK (MEK) inhibitor [68]. Therefore, we can speculate that the upregulation of ERK signaling would be caused by some hidden mutations in the upstream of the Ras-Raf-MEK-ERK pathway.

The weakness of this study is that we performed the experiment with only one cell line. It is in question whether the main mechanism of the radio-resistance of RT-R-MDA-MB-231 cells can be applied to all radiation-resistant breast cancer cell lines or can be generalized to triple-negative breast cancer cells. In addition, even regarding the radio-resistance of RT-R-MDA-MB 231 cells, other signaling pathways are also suggested as a key signaling pathway involved in the resistance. Similar to the signaling involved in CSC, the signaling involved in the radio-resistance of RT-R-MDA-MB 231 cells could also be complexed. However, aberrantly upregulated ERK signaling contributes to cancer cell proliferation, survival, and metastasis [14], and many other reports have suggested that ERK signaling is an important signaling pathway in radio-resistance [5,15,52]. Therefore, further research is also warranted regarding ERK signaling on the radio-resistance of breast cancer, especially on TNBC.

## 5. Conclusions

In summary, we found that ERK signaling was highly activated in RT-R-MDA-MB-231 cells compared to p-MDA-MB-231 cells. The activated ERK signaling was associated with an increased cancer stemness and EMT phenotype. In addition, the RT resistance of RT-R-MDA-MB-231 cells was reversed by the inhibition of ERK signaling. Furthermore, the inhibition of ERK suppressed the CSC marker proteins. With all of these findings, we conclude that activated ERK signaling is one of the major hub signals related to the acquisition of radio-resistant MDA-MB-231 cells. This study suggests a distinct and advantageous therapeutic value of the targeting of the ERK signaling pathway in MDA-MB-231 cells. Further research is also warranted regarding ERK signaling on the radio-resistance of breast cancer, especially on TNBC.

## Figures and Tables

**Table 1:** GO enrichment analysis of differentially expressed proteins. GO enrichment analysis showed that MAPK1 is related to all the suggested signaling pathways involved in the radio-resistance of RT-R-MDA-MB-231 cells.

Pathways	Genes	Genes Involved in the Pathways (%)	KEGG Pathway Entry
MAPK signaling pathway	MAPK1, CASP3, FGF22, MAPK11, IL1A	18	map04010
NOD-like receptor signaling pathway	MAPK1, HSP90AA1, MAPK11	15	map04621
PI3K-Akt signaling pathway	MAPK1, EIF4EBP1, HSP90AA1, FGF22, F2R	15	map04151
Pathways in cancer	MAPK1, CASP3, HSP90AA1, FGF22, F2R	13	map05200
Rap1 signaling pathway	MAPK1, FGF22, MAPK11, F2R	13	map04015
TNF signaling pathway	MAPK1, CASP3, MAPK11	11	map04668
Proteoglycans in cancer	MAPK1, CASP3, MAPK11	8	map05205
Regulation of actin cytoskeleton	MAPK1, FGF22, F2R	7	map04810

**Table 2:** List of studies involving radiation resistance and sensitivity in MDA-MB-231 cells or TNBC, and their possible mechanisms behind it. The reports were mainly categorized into three parts as follows: (i) specific signaling pathways involved in radio-resistant breast cancer cells, (ii) signaling pathways involved in radio-sensitivity, (iii) altered expression of gene/proteins involved in radio-resistant breast cancer cells. (iv) Studies that did not fall into any of the three above categories. Abbreviations: IFIT2—interferon-induced protein with tetratricopeptide repeats 2, TRIB3—tribbles homolog 3, ESM-1—endothelial cell-specific molecule-1, DLX2—vertebrate distal-less homeobox 2.

No.	Author	Key Findings	Year	References
(i) Specific signaling pathways involved in radio-resistant breast cancer cells				
1.	Gray et al.	The radiation-resistant ER+ breast cancer cell line (MCF-7, ZR-751) showed increased migration and invasion compared to the radiation-resistant ER- breast cancer cell line (MDA-MB-231). ER+ cells also showed a shift from ER to EGFR signaling pathways with increased MAPK and PI3K activity.	2019	[26]
2.	Ediriweera et al.	A phenolic lipid, 10-Gingerol, promotes apoptosis in radiation-resistant MDA-MB-231 cells through the PI3K/Akt signaling pathway.	2020	[27]
3.	Jin et al.	The overexpression of ESM-1 plays a critical role in radiation-resistant MDA-MB-231 cells through the regulation of PDK, PKC, and ERK1/2 pathways, and the subsequent activation of transcription factors HIF-1α, NF-κB, and STAT-3 to regulate adhesion molecules, MMPs, and VEGF.	2020	[28]
4.	lu et al.	The Wnt/β-catenin signaling pathway plays an important role in the development of radioresistance and Niclosamide, an FDA-approved anthelmintic drug that induces radiosensitivity in radiation-resistant MDA-MB-231 via inhibiting STAT3 and Bcl-2.	2018	[29]
5.	Bravatà et al.	Gene expression profiles of the MDA-MB-231 radiation cell fraction show increased TNF signaling, Phagosome, NF-kappa B signaling, Jak-STAT signaling, and PI3K-Akt signaling.	2019	[30]
6.	Choi et al.	DLX2 expression with irradiation incidence causes the increase in the EMT process and CSCs population through the Smad2/3 signaling pathway in MDA-MB-231 and A549 cells.	2016	[31]
(ii) Signaling pathways involved in radio sensitivity of MDA-MB-231 cells				
7.	Yin et al.	Niclosamide, an antihelminthic drug, inhibited the Wnt/β-catenin signaling pathway and increased the radiation sensitivity to triple-negative breast cancer (TNBC) cells (MDA-MB-231, MDA-MB-468, and Hs578T cells).	2016	[32]
8.	Lin et al.	COX-2 upregulation promotes radioresistance in MDA-MB-231 cells through the p38/MAPK-mediated alteration of apoptosis and metastasis.	2013	[33]
9.	KO et al.	Radiation-resistant MDA-MB-231 cells showed an increased cell proliferation, cell adhesion, EMT process, and increased stem cell population.	2018	[8]
10.	Koh et al.	Baicalein reduced the stem cell-like properties and metastasis in radiation-resistant MDA-MB-231 cells through the upregulation of IFIT2.	2019	[34]
(iii) Altered expression of gene/proteins involved in radio-resistant MDA-MB-231 cells.				
11.	Lammering et al.	Irradiation increased the expression of the EGFR protein in MDA-MB-231 xenograft tumors.	2004	[35]
12	Kim et al.	Proteomic analysis revealed single and the fraction of radiation increased cathepsin D (CTSD), gelsolin (GSN), argininosuccinate synthase 1 (ASS1), and C-type mannose receptor 2 (MRC2) in MDA-MB-231 cells.	2015	[36]
13.	Miao et al.	Radiation-resistant MDA-MB-231 and MCF-7 cells showed an altered expression of several members of the HSP70 and HSP40, subfamilies of HSPs, and an increased level of HSPB8, a target of NF-κB that could be responsible for the development of radioresistance.	2019	[37]
14.	Lee et al.	Increased expression of TRIB3 in radiation-resistant MDA-MB-231 cells causes the resistance and knockdown of TRIB3 sensitized toward radiation.	2019	[38]
15.	HOU et al.	Microarray analysis of radiation-resistant MDA-MB-231 cells showed increased cell adhesion and EMT factors.	2019	[39]
16.	Yang et al.	Overexpression of a small RNA molecule miR-634 decreases the survival rate of radiation-resistant MCF-7 and MDA-MB-231 cells by direct interaction with STAT3.	2020	[40]
(iv) Studies that did not fall into any of the three above categories.				
17.	Li et al.	A small molecule, ABT-787, induces radiosensitivity in radiation-resistant MDA-MB-231 by targeting Bcl-2 and Bcl-xL.	2012	[41]
18.	Nguyen et al.	A phytochemical phenethyl isothiocyanate reduces the CSC population in radiation-resistant MDA-MB-231 cells through upregulating ROS levels and targeting Metadherin at the post-transcriptional levels.	2020	[42]
19.	Oommen and Prise	A novel benzylidene lactam compound, KNK437, inhibits HIF-1α, HSF1, and AKT in hypoxia-induced MDA-MB-231 and T98G cells, which, in turn, induces radiosensitivity.	2012	[43]
20.	Kuger et al.	PI3K/mTOR inhibitor NVP-BEZ235 showed a synergistic effect with irradiation (IR) in MCF-7 and MDA-MB-231 cells.	2014	[44]
21.	Holler et al.	The molecular targeting of Akt by Akt inhibitor MK2206 or the knockdown of Akt1 led to a rapamycin-induced radiosensitization of SK-MES-1, HTB-182, or MDA-MB-231 cells by increasing DNA-double-stranded breaks.	2016	[45]
22.	Chen et al.	The estrogen receptor mediates the radiosensitivity of TNBC cells.	2017	[46]
23.	Liu et al.	Hypoxia due to a high cell density downregulated the EGFR expression and increased the sensitivity to ionizing radiation in MCF-7 and MDA-MB-231 cells.	2018	[47]
24.	Arnold et al.	STAT3 inhibition combined with radiation reduces the cellular plasticity in MDA-MB-231 and SUM159PT cells.	2019	[48]

## Data Availability

Samples are not available from the authors.

## Abbreviations

CSCs	Cancer stem cells
CypA	Cyclophilin A
AIF	Apoptosis-inducing factor
EMT	Epithelial-to-mesenchymal transition
ERK	Extracellular-signal-regulated kinase
DDR	Damage response
IR	Irradiation
PI3K	Phosphatidylinositol 3-kinase
MAPK	Mitogen-activated protein kinase
Wnt	Wingless-related integration site
GO	Gene Ontology
KEGG	Kyoto Encyclopedia of Genes and Genomes
STRING	Search Tool for the Retrieval of Interacting Genes
DMSO	Dimethyl sulfoxide is an organosulfur
MTT	3-(4,5-dimethylthiazol-2-yl)-2,5-diphenyltetrazolium bromide
PBS	Phosphate-buffered saline
DAPI	4′,6-diamidino-2-phenylindole
SDS-PAGE	Sodium dodecyl sulfate-polyacrylamide gel electrophoresis
SDS	Sodium dodecyl sulphate
RT	Radiotherapy
RT-R	Radiotherapy-resistant
SD	Standard deviation
CLK1	CDC-like kinases
FGF22	Fibroblast growth factor 22
F2R	Coagulation factor II (thrombin) receptor
Gy	Gray
CCD	Charge-coupled device
JAK2	Janus-activated kinase 2
PI3K-Akt	Phosphatidylinositol 3-kinase (PI3K)-protein kinase B (AKT)
STAT	Signal transducer and activator of transcription
NF-κB	Nuclear factor-κB
EGFR	Epidermal growth factor receptor
SOS	Son of Sevenless
B-raf	B-Rapidly Accelerated Fibrosarcoma
Ras	Rat sarcoma
MEK	Mitogen-activated protein kinase kinase
CASP9	Caspase 9
IFIT2	interferon-induced protein with tetratricopeptide repeats 2
TRIB3	Tribbles homolog 3
ESM-1	Endothelial cell-specific molecule-1
DLX2	Vertebrate distal-less homeobox 2
